# A Technique for Measuring Petal Gloss, with Examples from the Namaqualand Flora

**DOI:** 10.1371/journal.pone.0029476

**Published:** 2012-01-10

**Authors:** Heather M. Whitney, Sean A. Rands, Nick J. Elton, Allan G. Ellis

**Affiliations:** 1 School of Biological Sciences, University of Bristol, Bristol, United Kingdom; 2 Centre for Behavioural Biology, School of Veterinary Sciences, University of Bristol, Bristol, United Kingdom; 3 Interface Analysis Centre, University of Bristol, Bristol, United Kingdom; 4 Botany and Zoology Department, Stellenbosch University, Stellenbosch, South Africa; University of Northampton, United Kingdom

## Abstract

The degree of floral gloss varies between species. However, little is known about this distinctive floral trait, even though it could be a key feature of floral biotic and abiotic interactions. One reason for the absence of knowledge is the lack of a simple, repeatable method of gloss measurement that can be used in the field to study floral gloss. A protocol is described for measuring gloss in petal samples collected in the field, using a glossmeter. Repeatability of the technique is assessed. We demonstrate a simple yet highly accurate and repeatable method that can easily be implemented in the field. We also highlight the huge variety of glossiness found within flowers and between species in a sample of spring-blooming flowers collected in Namaqualand, South Africa. We discuss the potential uses of this method and its applications for furthering studies in plant-pollinator interactions. We also discuss the potential functions of gloss in flowers.

## Introduction

The plant surface has a wide range of roles [1]. As a result many of the structures produced by the plant surface are multifunctional, and may be involved in interactions with both the biotic and abiotic environment. This has been well demonstrated, for example, in trichomes, which can act to reflect damaging ultraviolet radiation, reduce water loss by influencing the boundary layer, moderate temperature excesses, as well as acting as important anti-herbivory devices [2], [3].

This multifunctional property of the plant surface has also been found in the flower, where the structure of the epidermal cells can influence both biotic and abiotic features. For example, conical cells are typical floral surface structures found on almost 80% of flowering plants [4] which influence floral temperature [1], colour [5] and wettability [6] and also impact on pollinator foraging efficiency, and thereby pollinator preference, by enhancing the grip of pollinators on the petal [7], [8]. The floral surface can also directly influence pollinator perception, as cuticular striations on the epidermal surface can, independently of any pigment colour, generate structural colour [9].

However, there are still many features of the floral surface that are yet to be investigated either in terms of ecology, phylogenetic distribution or their biotic and abiotic roles. One of these features is floral gloss, where gloss is defined as the specular reflection of light from the surface of an object [10]. In plants, the plant surface gloss, either on petal or leaf, will be determined primarily by two factors: the refractive index of the outermost layer of the epidermis (the waxy cuticle in higher plants) and the surface structure [11]. The chemical composition of the cuticle waxes will determine the refractive index and will therefore have a direct impact on surface gloss [12]. While there have been no studies investigating the impact of surface structures directly on gloss, it has been shown that surface structures such as trichomes, salt bladders or a thick layer of wax crystals can increase the light reflected from a leaf by 20%-50% [11]. However, it is unknown whether this reflection is specular (and will therefore impact on surface gloss) or diffuse (where it will not). These surface properties can either be measured independently or as a single measure of gloss. The refractive index of the plant epidermis is usually measured using an integrating sphere [11], while the structure of the plant surface is frequently observed by Scanning Electron Microscopy [1], [4]. Surface gloss in petals has previously been measured using a sophisticated spectrophotometry system [13].

It has been long established that flower petals, even within the same genus, differ in the degree of displayed gloss [14]. However, neither the phylogenetic distribution of floral gloss nor its potential impacts have been investigated in any detail. One reason these studies may be lacking is that there is no simple, repeatable method of gloss measurement that can be used to study floral gloss in the field. Recently, methods have been described detailing the measurement of gloss in animals, where it has been shown that a high gloss surface can increase the conspicuousness of plumage and may also be associated with quality signalling [15], [16] These methods relied on the use of robust animal material in the form of bird feathers. Feathers, if preserved correctly, will maintain the same degree of structure and therefore of colour and gloss as a live bird. However, the same cannot be said for plant material. A method of comparing the gloss characteristics of two members of the Ranunculaceae was developed by Galsterer *et al.* [13], but this method, while elegant and informative, requires a sophisticated setup that required a condenser-focused, filtered light source, a spectrophotometer with a photomultiplier and a graduated translational-rotational stage that is not compatible with use in the field. This means that, except under ideal conditions, the risk of previously-collected plant tissue not being in an optimal state for gloss measurements is a problem. Therefore, to quantify gloss in plants, a method is required that is not only highly accurate and repeatable, but is also portable enough to be implemented in the field, such that the plant surface can be measured *in situ* and therefore maintain its structural integrity. Here we present a method for doing this, and use it to demonstrate the variety of glossiness found in flowers. We discuss the potential uses of this method, and its applications for furthering studies in plant-pollinator interactions.

## Methods

### Study species

We developed our technique for measuring floral gloss using 10 common spring-flowering species from Kamieskroon, Northern Cape, South Africa. All plant material was collected with the permission of Northern Cape Nature Conservation. We then used the approach to survey differences in gloss at three hierarchical levels 1) within petals (ray florets) of a single flower, 2) between individuals within populations and 3) between species. *Arctotheca calendula* (L.) Levyns, *Dimorphotheca sinuata* DC., *Dimorphotheca tragus* (Aiton) B.Nord, *Gazania krebsiana* Less., *Grielum humifusum* Thunb., *Moraea miniata* Andrews, *Osteospermum pinnatum* (Thunb.) Norl., *Tripteris amplectens* Harv., *Tripteris hyoseroides* DC., and *Ursinia calenduliflora* (DC.) N.E.Br were surveyed between 25^th^ August and 3^rd^ September 2009.

### Gloss measurement

**Figure 1:**
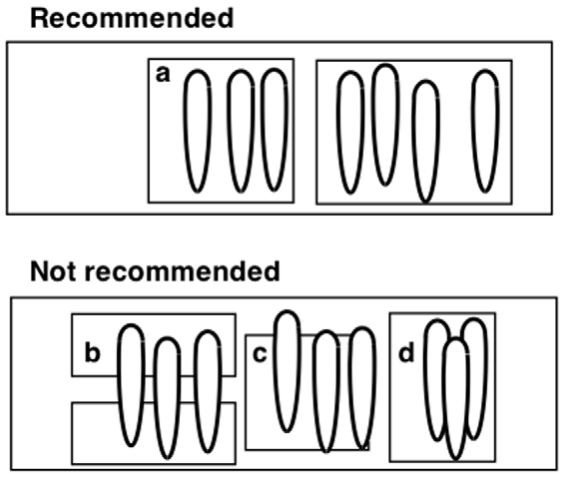
Recommendations for attaching petals to glass slides in preparation for gloss measurement.

Petals (or ray florets in the case of Asteraceae) were carefully removed from flowers, and gently stuck to a glass microscope slide using a layer of double-sided sticky tape. It is important that the petal is as flat as possible, and we strongly recommend that the area of tape used for attaching the petal is much larger than the petal itself (Figure 1*a*). Whilst conducting pilot work, we observed that using slivers of tape to stick down the ends of the petals (Figure 1*b*) or failing to stick the ends of the petal to the slide (Figure 1*c*) led to highly variable gloss measurements being recorded, and we therefore recommend ensuring that the petal is as flat as is physically manageable. We also recommend that petals are not allowed to overlap (Figure 1*d*), which can introduce unwanted texture and bending to the sample. In order to achieve the highest degree of flatness possible, we found that petals could be smoothed onto the double-sided tape with a soft paint-brush, creating as little physical damage to the petal surface as possible.

Gloss measurements were made using a ZGM 1120 Glossmeter (Zehntner Testing Instruments, Sissach, Switzerland), and recorded using the *GlossTools* 1.7 software supplied with the equipment, which was used to generate text files readable within standard spreadsheet software. This Glossmeter measures gloss by recording the light reflected at 20°, 60° and 85° away from the perpendicular to the surface, and compares this to a calibration standard (where 100% gloss is obtained from a black polished glass standard with a refraction index of 1.567 at a wavelength of 589.3 nm, supplied with the meter). Petal surfaces are not particularly glossy in comparison to the materials normally measured with this equipment, and the manufacturers ideally recommend using the 85° measurement head for such material. However, this requires a measuring area of 15×2 mm, which is larger than most of the petals collected (we experimented with this, and found that it was difficult to avoid partly measuring the mounting medium as well as the petal). According to the manufacturer's literature, there was too little gloss present to make the 20° measurements viable, and we therefore used the 60° measurements throughout, which required a 4.7×2 mm aperture. It should be noted that the measurements we were taking were lower than recommended by the manufacturer for ideal measurement using this angle, but the repeatability (discussed below) suggests that this is a suitable technique for comparing samples, although it is recommended that the same piece of equipment is used for all measurements if a comparative study is being undertaken. It is also recommended that the equipment is standardised regularly (we did this every 100 measurements, and always standardised before starting measurement of a new species).

For ease, the Glossmeter was attached upside-down to a table using pressure-sensitive putty adhesive (although any stable horizontal surface would do – the Glossmeter is a highly portable piece of equipment that could be used in the field, and could for example be stuck to a field notebook or the laptop it is attached to, provided that the meter didn't experience movement or vibrations whilst taking measurements). The slide could then be placed and left in place over the measurement aperture without having to be touched during measurement. During measurement, it was possible to observe which region of the petal had been sampled, as some of the light produced by the Glossmeter passed through the sample rather than being reflected and measured. The surface that the petals were attached to had no effect upon the glossiness measured, because it is only the light reflecting off the exposed surface (the petal epidermis) that is being measured. During pilot studies we checked this by attaching the petals to a variety of different surfaces, which had no effect upon the glossiness measured. (We would recommend carefully checking this assumption if exceptionally thin or translucent petals are being studied in future.) We recommend glass slides covered with sticky tape here for convenience: the solid slides could be easily handled, and petals could be removed intact, meaning that a prepared slide could be used multiple times. Furthermore, using a transparent slide makes it easier to observe where the measurement spot is falling during data collection.

For each species measured, five pairs of petals were removed from different plants. One of each pair was stuck to a slide with the adaxial surface facing upward (referred to as the petal ‘front’ here), and the other was stuck with the abaxial surface facing upward (referred to as the petal ‘back’ here). Where possible, multiple petals were placed on the same slide (as in Figure 1*a*) for convenience. For each petal surface, we took measurements of the region near the apex (tip) of the petal, and near the base of the petal. This design allowed us to explore variability in gloss along petals, between front and back petal surfaces, between individuals within a species and between species. In order to check the repeatability of measurements, for each of the species examined we took five readings of each petal surface×position combination from each of five individuals. Measurements were made with the long axis of the light spot parallel to the longitudinal base-tip axis of the petals. Between each of these five readings, the slide was picked up and replaced in the same position.

### Spectrophotometry Measurements

To compare Glossmeter readings to those obtained using the angle-specific spectrophotometry approach of Galsterer *et al*. [13] we measured the spectral reflectance of two of the species surveyed (*O. pinnatum* and *D. sinuata*). As described above, petals were attached with double-sided tape to a glass slide. An *Ocean Optics* S2000 spectrometer (range of 250–880 nm, Dunedin, FL, USA) with a xenon light source provided via a fibre-optic cable at an angle of 45° to the horizontal sample surface was used to obtain reflectance spectra. The fibre-optic measuring probe was set such that measurements at both a ‘pigment’ (light source and probe at 45° to the horizontal) and ‘mirror’ (light source at 45° and probe at 135° to the horizontal) geometry were obtained from each specimen. Ambient light was excluded when measurements were taken. All reflectance data were generated relative to a white standard (WS-1, Ocean Optics, Dunedin, FL, USA). We used *OOIBase* software to record the spectra.

### Statistical analyses

In order to assess the repeatability of the technique, the gloss measurements for all ten species were considered together. Considering the five measurements taken of each petal spot to be the replication level of interest, we assessed repeatability using the *rpt.aov* function from *rptR* [17] within *R* 2.11.1 [18], using the cube root values of the gloss measurements in order to satisfy standard ANOVA assumptions.

For the gloss measurements recorded for each of the ten species, a repeated-measures ANOVA was conducted using the mean values of the five measures taken from each spot, which included side (front *versus* back of the petal), end (tip *versus* base of the petal), and the interaction of end and side. To satisfy test assumptions, the cube roots of the mean spot measurements taken for *A. calendula*, *D. sinuata* and *M. miniata* were used. All tests were conducted using *R* [18].

## Results

The spot measurements taken were highly repeatable (*R* = 0.954±0.005 SE, CI = (0.945, 0.964), *p*<0.001), suggesting that gloss measurements made using the described technique were extremely reliable.

**Figure 2:**
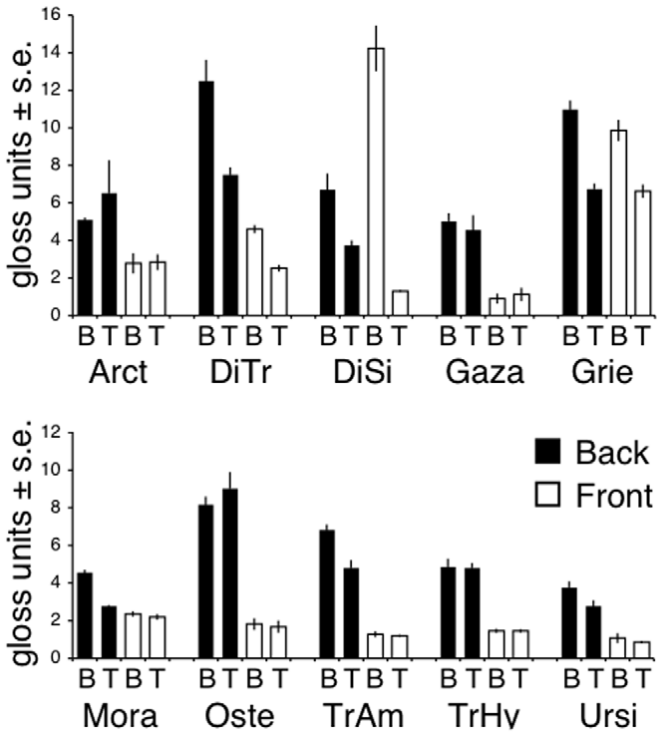
Gloss measurements. Gloss measurements at 60° angle, for the base (B) and tip (T) of the front and back of petals taken from: Arct, *Arctotheca calendula*; DiTr, *Dimorphotheca tragus*; DiSi, *D. sinuata*; Gaza, *Gazania krebsiana*; Grie, *Grielum humifusum*; Mora, *Moraea miniata*; Oste, *Osteospermum pinnatum*; TrAm, *Tripteris amplectens*; TrHy, *T. hyoseroides*; Ursi, *Ursinia calenduliflora*.

**Figure 3:**
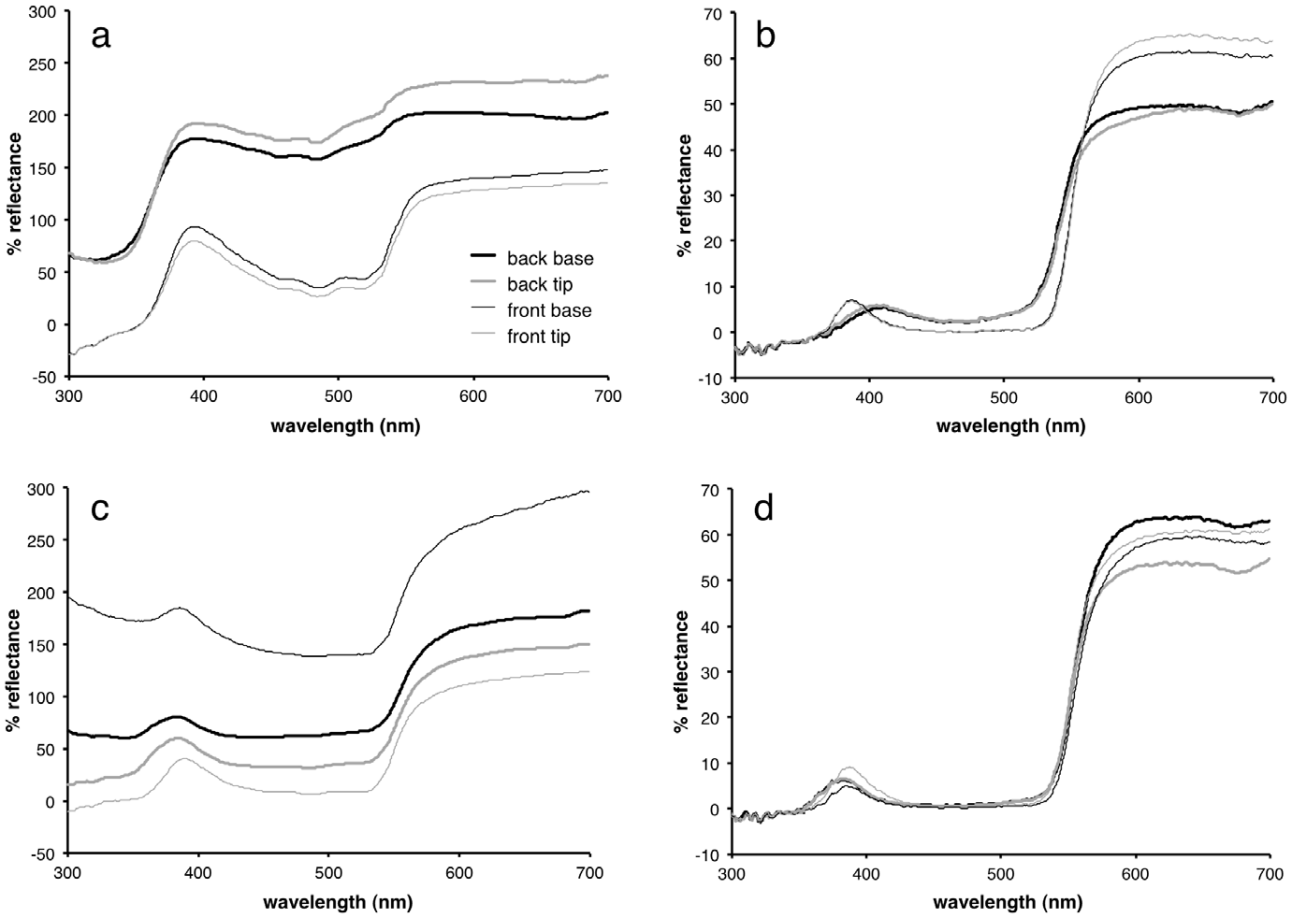
Reflectance curves. *a*) *Osteospermum pinnatum* mirror angle; *b*) *O. pinnatum* pigment angle; *c*) *Dimorphotheca sinuata* mirror angle; *d*) *D. sinuata* pigment angle. Thick lines denote the back of the petals, thin lines the front; black lines denote the base of the petals, grey lines denote the tips. Panels *a*, *b* and *d* are the mean values for three sets of measurements; *c* is the mean values for five sets of measurements.

Different species showed differing degrees of glossiness at both ends of both sides of their petals (Figure 2), with large, highly visible differences within some species (Table 1). The glossmeter results were confirmed by readings taken with the spectrophotometer, with highly glossy petal regions (for example the back of the *O. pinnatum* petals) showing a much higher percentage reflection at a specular angle than the less glossy regions (such as the front tip of the *O. pinnatum* petals) (Figure 3).

**Table 1:** ANOVA results.

	end	side	end×side
*Arctotheca calendula*	34.25**	105.11***	1.42^NS^
*Dimorphotheca sinuata*	269.93***	1.40^NS^	270.56***
*Dimorphotheca tragus*	105.12***	115.36***	7.25^NS^
*Gazania krebsiana*	0.72^NS^	21.04**	4.09^NS^
*Grielum humifusum*	47.24**	0.81^NS^	1.28^NS^
*Moraea miniata*	841.97***	80.53***	202.78***
*Osteospermum pinnatum*	0.05^NS^	107.28***	3.64^NS^
*Tripteris amplectens*	31.29**	174.11***	1.63^NS^
*Tripteris hyoseroides*	0.04^NS^	297.15***	1.46^NS^
*Ursinia calenduliflora*	11.11*	464.05***	16.50*

*F* and significance values for repeated-measures ANOVA results comparing gloss at the ends (tip or base) on different sides (front or back) of petals. All *F* values have 1, 4 degrees of freedom. Significances:

NS *p*≥0.05;

*p<0.05;

**p<0.01;

***p<0.001.

## Discussion

Here, we have demonstrated that the use of a Glossmeter in the field is an effective and repeatable method for the recording of floral gloss. This technique produces results with non-standard units of measurement (in gloss units, which are specific to a given angle of measurement), but our results show that it gives sufficiently quantified data to allow easy comparison within a flora (or in any other comparative framework). The technique described is fast, replicable, and requires little time or space to set up. Some spectrophotometric techniques can also be bought into the field, but only the use of a Glossmeter allows measurement of gloss without having to account for colour or other non-surface properties. Note that we have only assessed repeatability of measurement using a single piece of equipment here. As an industrial piece of equipment, the Glossmeter is designed to give highly repeatable measurements of non-biological surfaces (*e.g.* paint, plastic or metal) that are comparable between different meters, but biological surfaces are much less glossy than the optimal range measured by the meters. Therefore, we would recommend comparing measurements between meters (and between individual researchers using the same piece of equipment) before attempting meta-analyses across datasets.

Furthermore, this method has demonstrated that flowers show a diverse range of gloss levels on their petals, even when coming from within the same flora. Since a large amount of variation within flowers and between species demonstrably exists, should we therefore look a little deeper at where and why floral gloss occurs? Some hypotheses for some of the functions of gloss in floral tissue have previously been stated. Floral gloss may mediate plant-pollinator interactions. Galsterer *et al.* [13] mention that gloss has a dynamic component that will change with angle of light or insect approach, which may help visiting insects with long distance orientation as they approach the flower. Also, if specific portions of the flower are glossy, it has been suggested that this gloss could mimic nectar [19], and we suggest it could also mimic other resources collected by pollinators in this environment such as oil, moisture or wax.

Gloss could also enhance floral salience (the detectability of a flower by a pollinator). The specular reflection could result in a cue visible from a greater or distance, or one that in certain conditions could provide greater contrast with the surrounding foliage. Gloss is also structurally and optically linked with iridescence [20], which has been shown to increase floral salience [21]. However, these advantages of orientation and salience may come at a cost as, like iridescence, gloss could reduce colour constancy due to its dynamic nature [21].

A glossy surface may also have direct advantages to a flower that do not involve their pollinators. A glossy petal will reflect a greater proportion of the incident light. This could help control floral temperature, which could both reduce the desiccation risk and could also be of benefit when attracting pollinators, as floral temperature has been found to be a reward in itself [22]–[24]. Reflection of specific wavelengths could also be protective, by protecting the flower and the fragile reproductive structures from potentially damaging UV radiation.

Gloss may also be the inadvertent product of other floral properties, such as surface wettability, and as such could potentially be an easily measured initial indicator of these traits. Like gloss, plant surface wettability is affected both by the chemical composition and the structure of the plant cuticle [1]. However, because these two factors (surface chemistry and surface structure) are independent, changes in either of them could feasibly change surface gloss and wettability in different ways and to different extents. Similarly, the adhesion of herbivores to the plant surface is also affected by both cuticle chemistry and structure [25]. Thus a change in either surface chemistry or structure could independently impact on a range of surface properties (including gloss) in ways that are difficult to predict without experimental investigation. The method described in this paper is sufficiently simple and versatile to render the possibility of a systematic study of these potential interactions possible.

There are many reasons why floral gloss may be an interesting feature of flowers that has been involved in plant-pollinator co-evolution. It has not been one that has been widely investigated, but we hope that this simple and repeatable method will contribute to exploring this little-studied floral feature.
